# Attenuation of osteoarthritis progression through intra-articular injection of a combination of synovial membrane-derived MSCs (SMMSCs), platelet-rich plasma (PRP) and conditioned medium (secretome)

**DOI:** 10.1186/s13018-021-02851-2

**Published:** 2022-02-17

**Authors:** Sara Sadat Nabavizadeh, Tahereh Talaei-Khozani, Moein Zarei, Shahrokh Zare, Omid Koohi Hosseinabadi, Nader Tanideh, Sajad Daneshi

**Affiliations:** 1 Student Research Committee, Shiraz University of Medical Sciences, Shiraz, Iran; 2 Department of Anatomical Sciences, School of Medicine, Shiraz University of Medical Sciences, Shiraz, Iran; 3 Laboratory for Stem Cell Research, Department of Anatomical Sciences, Shiraz University of Medical Sciences, Shiraz, Iran; 4 Department of Polymer and Biomaterials Science, Western Pomeranian University of Technology, Szczecin, Al. Piastow 45, 71-311 Szczecin, Poland; 5 Stem Cells Technology Research Center, Shiraz University of Medical Sciences, Shiraz, Iran; 6 Central Research Laboratory, Shiraz University of Medical Sciences, Shiraz, Iran; 7 Department of Pharmacology, Medical School, Shiraz University of Medical Sciences, Shiraz, Iran

**Keywords:** Osteoarthritis, Mesenchymal stem cells, PRP, Secretome, Rat

## Abstract

**Purpose:**

Osteoarthritis (OA) as a progressive destructive disease of articular cartilage is the most common joint disease characterized by reduction of joint cartilage thickness, demolition of cartilage surface and new bone formation. To overcome these problems, the purpose of the current research was to evaluate and compare the in vivo effects of synovial membrane-derived mesenchymal stem cell (SMMSCs), platelet-rich plasma (PRP) and conditioned medium (secretome) on collagenase II-induced rat knee osteoarthritis (KOA) remedy.

**Methods:**

For the first step, SMMSCs were isolated and characterized. Also, secretome was collected from SMMSCs culture. Furthermore, PRP was collect from the rat heart venous blood. Second, two injection of collagenase II with an interval of 3 days was performed in the knee intra-articular space to induce osteoarthritis. Two weeks later, animals were randomly divided into 6 groups. Control group without treatment, positive group: taken an intra-articular sodium hyaluronate injection (0.1 ml), treatment groups taken an intra-articular injection of; treatment 1: SMMSCs (5 × 10^6^), treatment 2: SMMSCs (5 × 10^6^)/secretome (50 µl), treatment 3: SMMSCs (5 × 10^6^)/PRP (50 µl), and treatment 4: SMMSCs (5 × 10^6^)/ secretome (50 µl)/ PRP (50 µl). Three months later, rats were killed and the following assessments were executed: radiography, histopathology, and immunohistochemistry.

**Results:**

Our findings represented that a combination of the SMMSCs/secretome/PRP had a considerable effect on glycosaminoglycans (GAGs) and collagen II contents, articular cartilage preservation, compared with other groups. In addition, combination of the SMMSCs with PRP and secretome showed the lowest expression of mmp3, while SOX9 had the highest expression in comparison with other groups. Also, SMMSCs-injected groups demonstrated better results compared with positive and control groups.

**Conclusions:**

Injecting a combination of the SMMSCs/secretome/PRP resulted in better efficacy in terms of joint space width, articular cartilage surface continuity and integrity, sub-chondral bone and ECM constituents such as collagen II. Indeed, transplantation of this combination could be considered as a preliminary therapy for clinical trial study in the future.

## Background

Osteoarthritis (OA) as a polygenic disease is a debilitating, irreversible, degenerative, and severe joints disorder like in the hip, knee, and hand in humans. OA associates with articular cartilage demolition and decrement, hypertrophy of the synovium, alterations in the ligaments, capsule and synovial membrane, osteophytes formation or aberrant bone outgrowths, and increased thickness of the subchondral bone in which leads to alleviate daily living activity [1–4]. Universally, over 250 million patients are suffered from the OA especially knee osteoarthritis (KOA) whereby 30% of older people aged more than 60 years have gait dysfunction, pain, instability, stiffness, and their joint space is declined [5, 6]. To date, different methods and materials have been applied in order to cure for KOA. In recent years, an increasing number of experiments have shown that current procedures such as exercises, medications, physical therapy, and surgery have not been fully successful in treatment of KOA and prevention of damage to the joint tissue [7–13]. Furthermore, knee joint replacement as a gold procedure to cure KOA is painful and expensive along with inflammation and bleeding. Taking all these problems and limitations into consideration, an outlook of therapy on future has been focused on stem cells and biological products [12, 14].

A multitude of studies have applied mesenchymal stem cells (MSCs) as a potent tool to cure different diseases. Their characteristics like self renewably, anti-inflammatory and immunomodulatory effects have made them as a promising option for treatment of diseases [15–17]. Moreover, MSCs, multipotent stromal cells, can differentiate into multiple lineages of cells including chondrocytes (cartilage) and osteoblasts (bone) whereby limitations of poor intrinsic regeneration of cartilage tissue lesion due to the disability of resident articular chondrocytes to secrete a matrix could be separated [18, 19]. Derived-MSCs of various sources such as amniotic fluid, bone marrow, umbilical cord, adipose tissue, synovial fluid have been evaluated in a wide range of different experimental disease models [20]. In addition to general properties of MSCs, their paracrine activity, angiogenesis and chondrogenic potential made stem cell therapy an interested subject for treatment of KOA wherein extracellular matrix is damaged and chondrocyte function decreased [21, 22].

Previous studies have shown that post-intra-articular injection of MSCs, their proliferation decreased and differentiation into cartilage cells impaired. Nonetheless, it is notable that conditioned medium of MSCs known as secretome or MSCs paracrine is containing hormones, signaling biomolecules, chemokines, extracellular vesicles, growth factors, and cytokines affecting host cells functions such as differentiation, immigration, and secretion. Besides, secretome has simply freezing which helps it to be conserved for a long time [4, 23, 24].

Platelet-rich plasma (PRP) as an autologous blood derived product without transfer of blood diseases and immunological reactions is safe [25]. Also regarding the treatment of KOA, it is reported that PRP has similar effects like intra-articular injection of hyaluronic acid. In addition, PRP, a volume of plasma with a platelet concentration (three to fivefold), contains growth factors such as platelet-derived growth factor (PDGF) and transforming growth factor **(**TGF-β), which in turn can stimulate responsible cells to repair and regenerate destructed cartilage and bone tissues in KOA patients which make it promising option. PRP has also been revealed to reproduce hyaluronan synthesis, enhance angiogenesis, and moderate inflammation [26].

Hence, to achieve the desired design targets, we have attempted to comparatively survey the effectiveness of the synovial membrane-derived MSCs, PRP and secretome in KOA betterment.

## Materials and methods

### Isolation and culture of rat synovial membrane-derived MSCs (SMMSCs)

The current research was performed at Center of Comparative and Experimental Medicine, Shiraz University of Medical Sciences, Shiraz, Iran. Adult male Sprague Dawley rats were killed in a CO_2_ chamber. Under sterile conditions, the skin and muscles of the knee were detached, the knee joint was exposed, ligaments were excised and synovial membrane was harvested from the inner side of the articular capsule. Subsequently, synovial membrane was transferred in a tissue plate, washed using sterile phosphate buffered saline (PBS; Gibco, USA) to eliminate unwanted tissues and minced into small pieces. Thereupon, 1 mg/ml Collagenase IA (Sigma-Aldrich, USA) was added to digest the explanted tissues at 37 °C, 5% CO_2_ for 1 h. Next, DMEM (Bioidea, UT, Iran) containing 10% heat inactivated fetal bovine serum (FBS, Bioidea, UT, Iran) was added to stop the digestion. Then, the digested cells were filtered using a 70-µm nylon filter to yield single-cell suspension and centrifuged at 1200 rpm for 15 min. Afterward, the cell pellet was resuspended in a growth medium containing Dulbecco’s modified Eagle’s medium (DMEM) low glucose, 10% fetal bovine serum (FBS; Gibco, USA) and 100 U/mL penicillin–streptomycin (Gibco, USA) in a 25-cm^2^ culture flask and incubated in 5% CO_2_ and 95% air at a temperature of 37 °C. After 48 h, the medium was gently removed and fresh medium was added every 3–4 days until 80–90% confluency, and then passaging to expand the MSCs population.

Thereafter, the cells were washed using PBS, treated with 0.5% Trypsin–EDTA (Gibco, USA) for 3 min to detach the adherent cells. Then, the same amount of culture medium was added to inactive the enzyme. The cell suspension was centrifuged at 1200 rpm for 5 min, the supernatant removed, and the cell pellet cultured in a 75-cm^2^ culture flask containing fresh medium. Cell culture was continued up to passage 3 while for characterization and intra-articular injection. [27].

### Phenotypic characterization

The procedure for flow cytometry was performed based on previously published method [28]. Cells at passage 3 were suspended in the PBS containing 2% FBS. Then, cold PBS containing 10% FBS as a blocking solution was used to wash a density of 1 × 10^6^ cells/mL of the cell suspension; for 20 min. Next, a density of 5 × 10^5^ cells was applied per each flow cytometry tube. The cells were labeled with ready to use FITC (fluorescein isothiocyanate)-conjugated anti-CD44, anti-CD45, PE (phycoerythrin)-conjugated anti-CD34, and PerCP (Peridinin Chlorophyll Protein complex)-conjugated anti-CD90 antibodies (all from Abcam, UK, Cambridge). Thereafter, the cell samples were incubated for 25 min in the dark at room temperature. Afterward, the cells were washed with PBS, centrifuged at 300*g* for 10 min, fixed with 1% paraformaldehyde for 15 min and finally washed with cold PBS. Then, the samples were resuspended in PBS containing 10% FBS, and analyzed by a flow cytometer (BD FACSCalibur, flow cytometer, USA). The data were then analyzed by FlowJo software (TreeStar, Ashland, OR, USA).

Furthermore, multi-potentiality was assessed by in vitro adipogenic and osteogenic differentiation.

#### Assay of multi-potentiality adipogenesis

SMMSCs at passage 3 were incubated in adipogenic media to differentiate into adipocyte for 2 weeks. In addition, a control media containing SMMSCs at passage 3 was preserved. The control medium consisted of DMEM (Gibco, USA), supplemented with 10% FBS (Gibco, USA) and 1% penicillin/streptomycin (Gibco, USA). The adipogenic medium contained DMEM, 15% FBS, 100 μM L-ascorbic acid, 200 μM indomethacin, 1000 nM insulin (Sigma-Aldrich), and 100 nM dexamethasone (Sigma-Aldrich). The medium was substituted 2 times a week. Finally, the cultured cells were fixed in 4% paraformaldehyde (PFA) for 20 min. Next, Oil Red-O Solution was applied to stain lipid vacuoles for 20 min at room temperature [29].

### Assay of multi-potentiality osteogenesis

SMMSCs at passage 3 were incubated in osteogenic media to differentiate into osteoblasts for 2 weeks. In addition, a control media containing SMMSCs at passage 3 was preserved. The control medium contained DMEM (Gibco, USA), 10% FBS (Gibco, USA) and 1% penicillin/streptomycin (Gibco, USA). The osteogenic medium consists of DMEM, 15% FBS (Gibco, USA), 100 μM L-ascorbic acid, 10 mM glycerol 3-phosphate, and 100 nM dexamethasone (Sigma-Aldrich). The medium was changed 2 times a week. Eventually, SMMSCs were fixed in paraformaldehyde (4%) for 20 min and then stained with Alizarin Red solution for 20 min at room temperature to stain calcium deposits [29].

### Conditioned medium (secretome) preparation

Tissue-culture dishes (150 mm) were used to SMMSCs culture at 37 °C with 5% CO_2_ and an atmosphere of 95% air. As the cell culture in DEMD became confluent, PBS was applied to rinse SMMSCs and the growth medium was substituted with DMEM. After 24 h, the conditioned medium was collected and centrifuged twice, first at 500×*g* for 10 min and afterward at 3000×*g* for 20 min, to remove cell debris. Finally, pre-cleaned secretome was concentrated to ~ 1.5 mL using Centriprep YM-3 centrifugal units (Millipore, Bedford, MA, USA) [30].

### Platelet-rich plasma (PRP) preparation

Animals were anesthetized with 100 mg/kg ketamine 10% (Alfasan, Netherlands) and 10 mg/kg xylazine 2% (Alfasan, Netherlands). Then, venous blood was drawn from the heart, poured in a citrated dextrose tube and centrifuged at 319×*g* for 2 min. The Supernatant layer, plasma fraction, composed of three distinct layers as follow: the upper layer was platelet-poor plasma (PPP), the middle layer was plasma average platelet (PAP) and the lower was platelet-rich plasma (PRP). The two top layers (PPP and PAP) were discarded by pipette. The third layer (PRP) was carefully separated by pipette and re-centrifuged at 319 × g for 5 min. Eventually, the plasma layer (upper layer) was separated and the PRP layer (second layer) was filtered to discard the leukocytes and then preserved for intra articular injection [31].

### Animal housing

Thirty-six adult male Sprague Dawley rats (200 ± 20 g body weight) were used in the current research. All animals were kept in standard cages with 55 ± 5% relative humidity, a 12-h light/dark cycle, and 22 ± 2 °C temperature. All animals had free access to water and food ad libitum. The rats were randomly divided into 6 groups (*n* = 6). This study followed the internationally accredited guidelines with ethical approval from the Institutional Animal Care and Use Committee of Transgenic Research Center of Shiraz University of Medical Sciences (Shiraz, Iran). The registration number was 97-01-67-19128.

### Osteoarthritis induction

General anesthesia was induced in rats by 100 mg/kg ketamine 10% (Alfasan, Netherlands) and 10 mg/kg xylazine 2% (Alfasan, Netherlands), and then the rat knee was shaved. Four mg collagenase II (clostridium histolithicum; Sigma-Aldrich, St. Louis, MO, USA) dissolved in sterile PBS, and afterward was injected in the knee intra-articular space. After 3 days, injection was repeated [32].

### Animal grouping

Two weeks after the second injection of collagenase II in which caused to induction of OA, animals were grouped as follows:

OA group: did not receive any treatment.

Positive group: taken an intra-articular sodium hyaluronate injection (0.1 ml with a concentration of 20 mg) (Fidia, Abano Terme, Italy).

Treatment 1: taken an intra-articular injection of SMMSCs (5 × 10^6^).

Treatment 2: taken an intra-articular injection of SMMSCs (5 × 10^6^)/ secretome (50 µl).

Treatment 3: taken an intra-articular injection of SMMSCs (5 × 10^6^) / PRP (50 µl).

Treatment 4: taken a combination of an intra-articular injection of SMMSCs (5 × 10^6^)/secretome (50 µl)/ PRP (50 µl).

### Radiography evaluation

Three months after onset of treatment, rats were killed in a CO_2_ chamber, and thereupon radiography was taken from the lateral aspect of knee joint using radiographic equipment (AxiomMultix M Radiographic Unit, SiemensTM, Germany). Osteoarthritis in terms of presence of osteophytes, joint space narrowing, and subchondral sclerosis as Radiological grading system was assessed by a blinded radiologist [32, 33].

### Histopathological study

After sacrificing, the knee joints were harvested and fixed with 10% formalin solution for 48 h. Then, samples were dehydrated with ethanol, embedded in paraffin, and cut into 4 μm sections before staining. Hematoxylin and eosin (H&E; Leica, Germany), Masson's trichrome (Merck, Germany), Toluidine blue (Merck, Germany), safranin O (Merck, Germany) and alcian blue (Merck, Germany) stains were used to evaluate cartilage structure, collagen formation, glycosaminoglycans [Molecular Weight (305 g/moles)], sulfated glycosaminoglycans of proteoglycans [Molecular Weight (350 g/moles)], and glycosaminoglycans [Molecular Weight (1341 g/moles)], respectively. The International Cartilage Repair Society (ICRS) scores were used to evaluate the samples [32].

### Immunohistochemistry (IHC)

The samples were incubated in 3% H_2_O_2_ (Sigma-7722-84-1) in methanol for 10 min to block the endogenous enzyme. Then, to block non-specific binding sites, the samples were incubated in PBS containing 10% goat serum and 5% Bovine Serum Albumin (BSA). Subsequently, the samples were incubated in anti-Mmp3, -SOX9 and -collagen type II antibodies (Abcam PLC, Cambridge, MA, USA) at dilutions 1/100, 1/250 and 1/200, respectively. The sections were then incubated with radish peroxidase/streptavidin (1:10,000; Abcam, USA) at room temperature for 20 min. Finally, diaminobenzidine was added as the chromogen [34].

### Statistical analysis

The data were analyzed by Mann–Whitney U test using the GraphPad Prism 5.0 software (GraphPad, La Jolla, CA). A value of p < 0.05 was considered as statistically significant.

## Results

### SMMSCs identification

**Fig.1:**
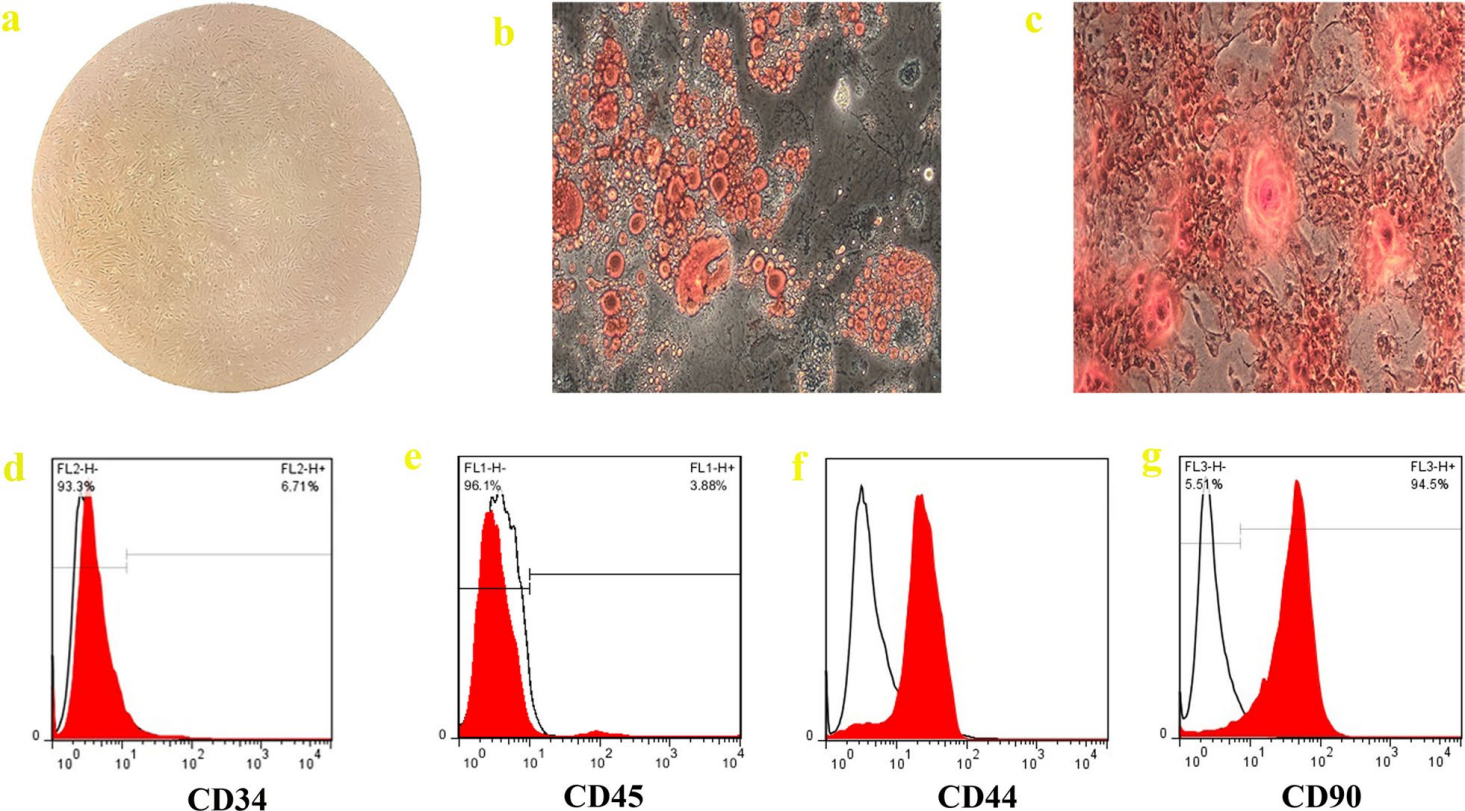
Morphology (**a**) (×10), differentiation capacity and cell surface markers of SMMSCs. For SMMSCs, adipogenic differentiation was shown by oil red staining (**b**), while osteogenic differentiation was demonstrated by calcium deposition in Alizarin red staining (**c**); ×10 magnification. Flow cytometry study findings exhibited that SMMSCs were negative for hematopoietic markers CD34 (**d**) and CD45 (**e**) and also positive for MSC markers CD44 (**f**) and CD90 (**g**)

Figure 1a shows the spindle-shaped morphology of SMMSCs at passage 3 that acquired from the synovial membrane of male Sprague Dawley rat. Oil red-O staining and alizarin red staining are used to demonstrate the differentiation capacities of SMMSCs to adipocyte and osteocyte, respectively. Oil Red-O staining showed of lipid-rich vacuoles after 2 weeks (Fig. 1b). In addition, Alizarin Red staining revealed the formation of mineralized nodules after 2 weeks (Fig. 1c). To characterize the phenotypes of SMMSCs, flow cytometry was executed to evaluate the surface markers of MSCs. Cells were labeled with FITC, perCP, and PE-conjugated antibodies and investigated by flow cytometry. Cells were stained with CD34, CD45 (hematopoietic cell markers) and CD44, CD90 (mesenchymal stem-cell markers). Based on our findings, the SMMSCs were negative for cell markers CD34, and CD45, while positive for MSC marker CD44 and CD90 (Fig. 1d–g).

### Histological assessments

#### H and E staining

**Fig.2:**
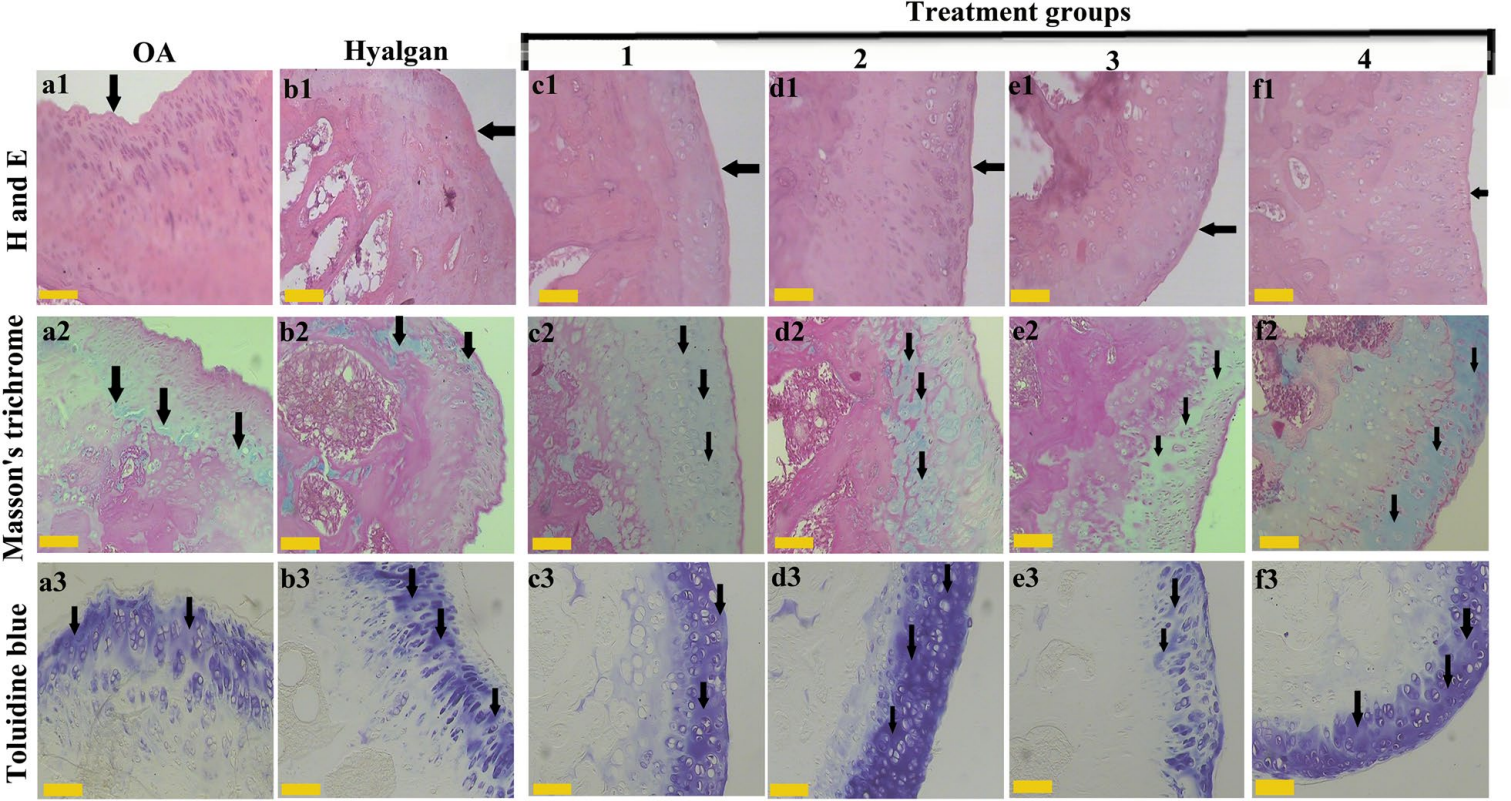
Different stains for knee articular cartilage structure and constituents. H and E staining showed better articular cartilage surface continuity (black arrow) in treatment groups (c1, d1, e1 and f1). Masson’s trichrome staining revealed more collagen type II (black arrow) (c2, d2, e2 and f2). Toluidine blue staining exhibited better extracellular matrix preservation (black arrow) in treatment groups (c3, d3 and f3). (The yellow scale bar shows 100 μm)

As revealed by the H and E staining, treatment groups (c1, d1, e1 and f1) had better articular cartilage surface continuity and integrity. More cell distribution was seen in treatment 4 group (f1), also, treatment 1, 2 and 3 groups (c1, d1 and e1) showed better cell distribution compared to OA (a1) and sodium hyaluronate (b1) groups. In addition, treatment groups (c1, d1, e1 and f1) demonstrated better cartilage mineralization compared to OA (a1) and sodium hyaluronate (b1) groups. Moreover, better subchondral bone was seen in treatment groups (c1, d1, and e1) especially in treatment 4 (f1). Besides, more cell population was observed in treatment groups (c1, d1, e1 and f1) especially in treatment 2 and 4 (d1 and f1). A regular and intact subchondral bone with normal interstitial space and lamellar orientations is seen in treatment groups (c1, d1 and f1), whereas treatment 3 (e1), OA (a1) and sodium hyaluronate (b1) groups showed destruction of trabecular bone (Fig. 2).

### Masson’s trichrome staining

**Fig. 3:**
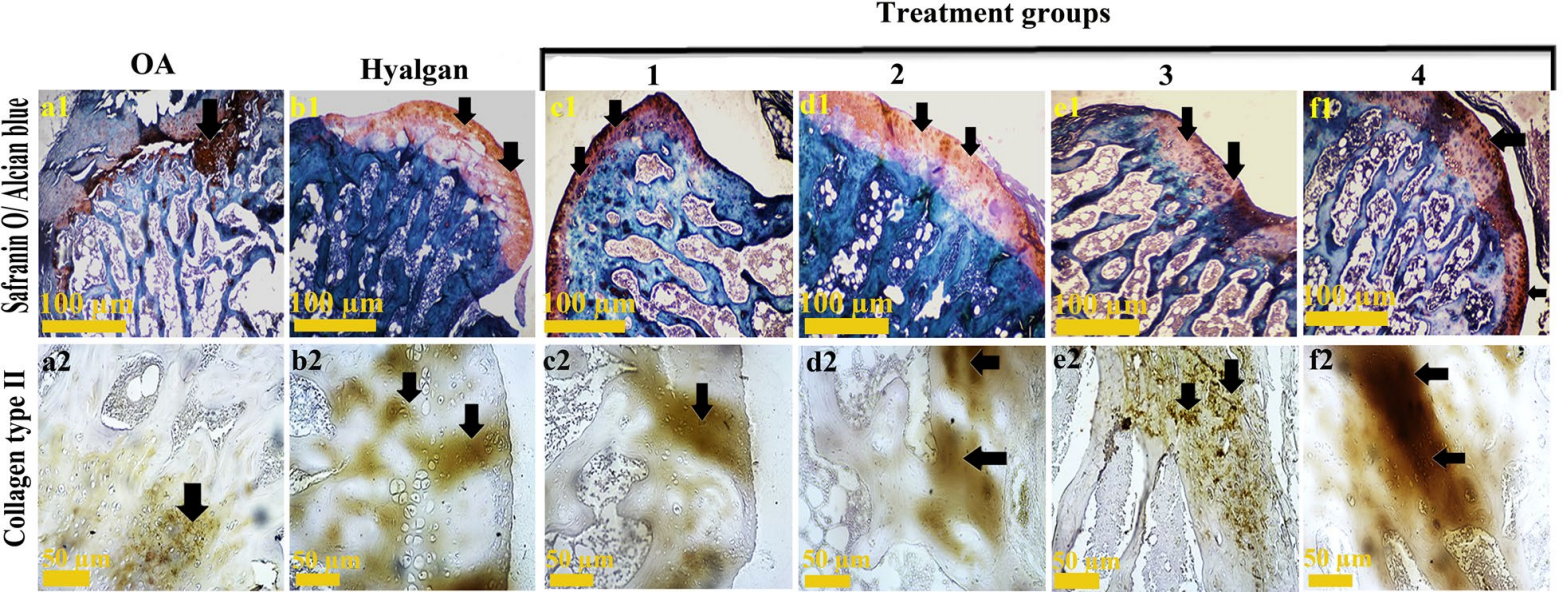
Special staining of knee articular cartilage. Safranin O/ alcian blue staining showed more GAGs (black arrow) in treatment groups c1, e1 and f1. Immunohistochemistry for Col2 (black arrow) revealed better expression in treatment groups (c2 and f2)

Those groups were received treatment groups (c2, d2, e2 and f2) exhibited more collagen type II using Masson’s trichrome staining. A potent orientation of collagen is seen at superficial layer of treatment 4 (f2), and deep layer of treatment 2 (d2). In addition, those groups receiving stem cells demonstrated regular surfaces, while loss of superficial cell layers and surface irregularities are observed in OA (a2) and sodium hyaluronate (b2) groups. Articular cartilage fraction and roughness were observed in some parts of e and f groups (Fig. 3).

### Toluidine blue staining

Following toluidine blue staining, better extracellular matrix and orientation of chondrocytes were observed in those animals administrated by treatment groups (c3, d3 and f3). The cartilage layer in treatment groups (c3, d3 and f3) was heavily stained in comparison with OA (a3).

### Safranin O/alcian blue staining

Based on safranin O/alcian blue staining, similar sulfated glycosaminoglycans of proteoglycan contents (red/orange) were observed in treatment groups (c1, e1 and f1) as the best findings. Likewise, treatment 2 (d1) and sodium hyaluronate (b1) groups were the same in glycosaminoglycans (GAGs) contents. Generally, all groups showed better results compared to OA group.

#### Immunohistochemistry assay

Based on IHC, better expression of Col2 was observed in treatment groups c2 and f2. Besides, similar Col2-positive cells were seen in treatment 2 (d2) and sodium hyaluronate (b2) groups.

**Fig. 4:**
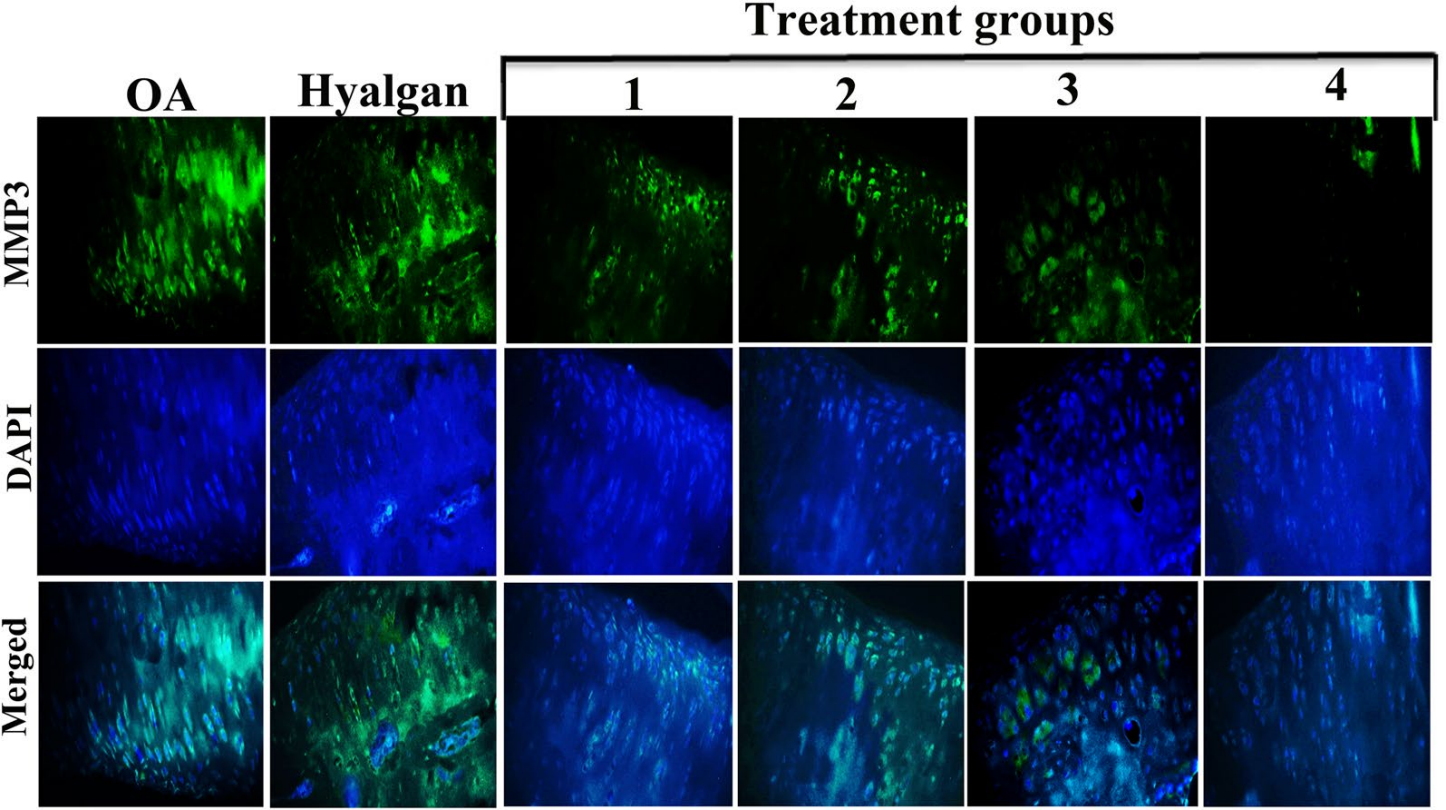
IHC evaluation of MMP3 in OA-induced knee articular cartilage. Compared with other groups, IHC revealed low expression of MMP3 in treatment group 4 (×10)

Lower expression of MMP3, as a proteolytic enzyme which is well known in the development and spread of inflammatory diseases such as osteoarthritis, was especially observed in treatment group 4 using IHC staining (Fig. 4). However, the level of MMP3 in OA and sodium hyaluronate groups was significantly increased. Treatment groups 1, 2 and 3 exhibited less expression of MMP3 compared with untreated groups (Fig. 4).

**Fig. 5:**
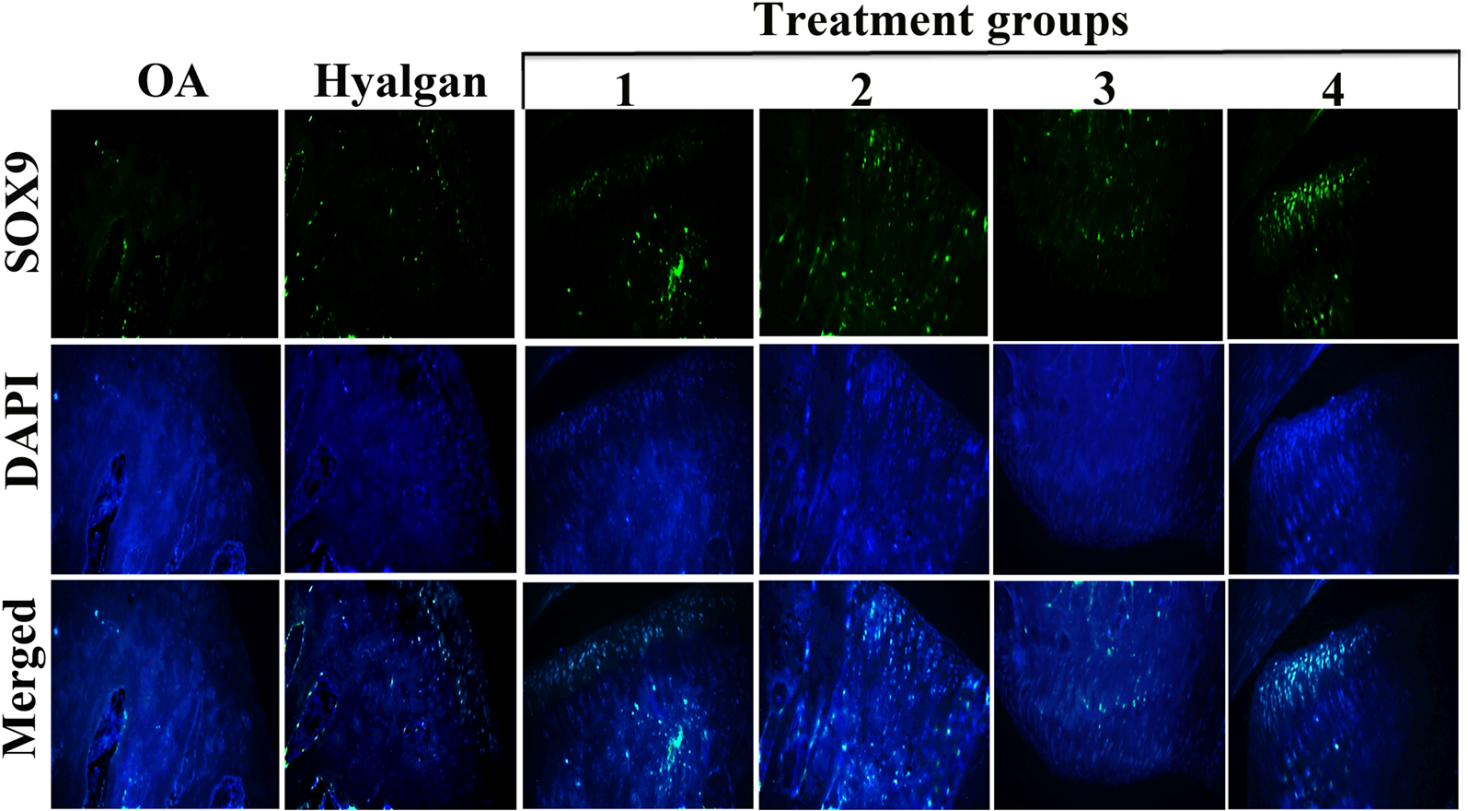
IHC evaluation of SOX9 in OA-induced knee articular cartilage. IHC revealed higher expression of SOX9 in treatment groups (2, 3 and 4) (×10)

To survey the potential effects of SMMSCs/secretome/PRP on OA-induced knee articular cartilage, IHC staining for SOX9 was done. Results showed higher expression of SOX9 in treatment groups 2, 3, and 4 (Fig. 5). Likewise, SOX9-positive cells were more observed in superficial layer of articular cartilage in treatment group 4 (Fig. 5).

### Radiography findings

**Fig. 6:**
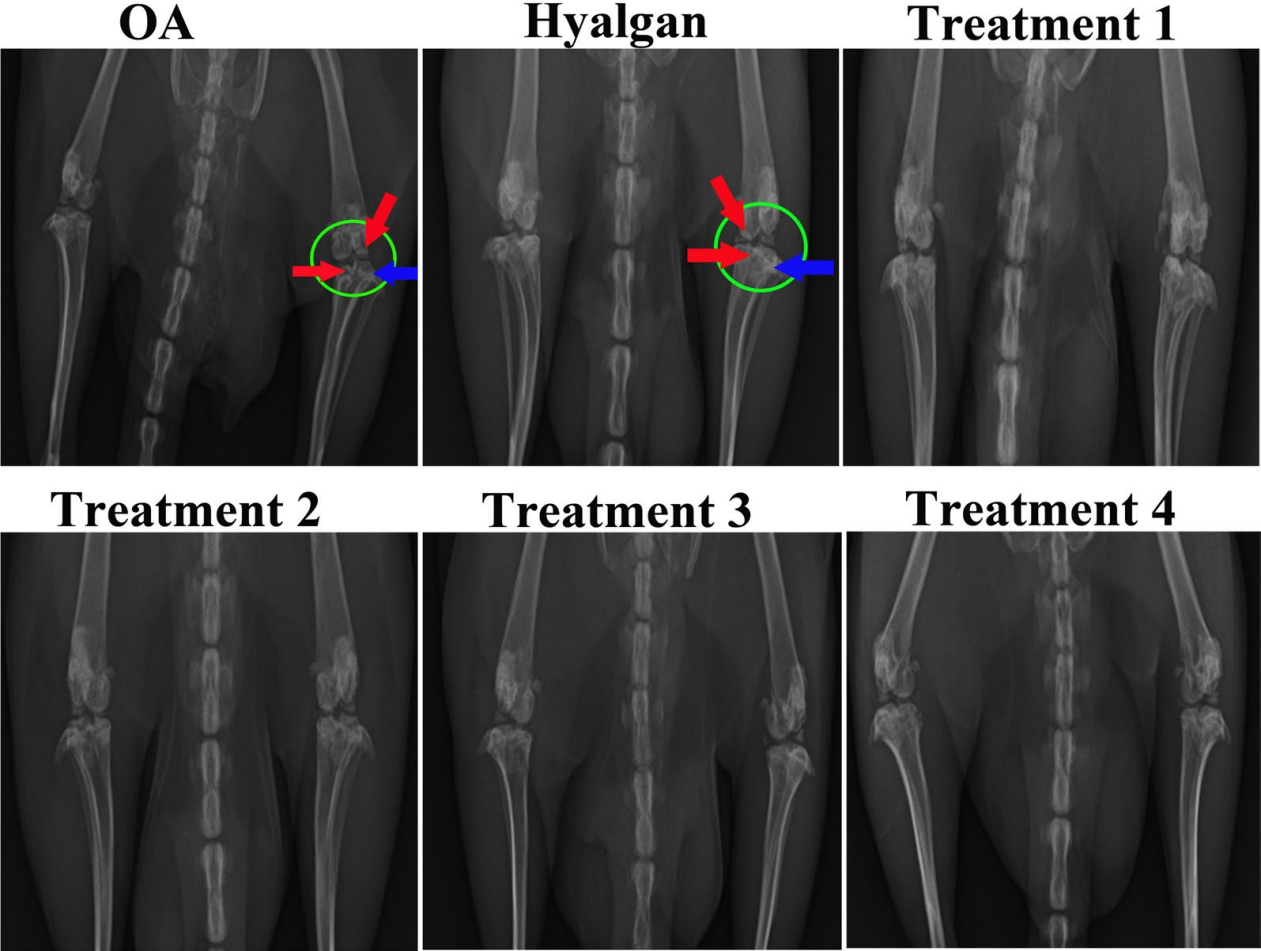
Radiography Study. Joint space narrowing (green circle), subchondral sclerosis (red arrow) and osteophyte formation (blue arrow) were observed in OA and Sodium hyaluronate groups

Joint space narrowing, subchondral sclerosis and osteophyte were not observed in treatment group 4. In treatment 1 and 2 groups, findings were normal but osteophyte was seen in early stage. A light Joint space narrowing and osteophytes in early stage were observed in treatment 3 group, while Joint space was normal. Joint space narrowing, moderate subchondral sclerosis and osteophyte were detected in OA and sodium hyaluronate groups, while osteophyte formation was more severe in OA group (Fig. 6).

## Discussion

This study demonstrated that intra-articular injection of SMMSC/secretome /PRP improves OA by enhancing cartilage regeneration. Previously, the individual effects of intra-articular injection of SMMSCs, PRP, and secretome for the treatment of collagenase-induced KOA in rat model were investigated, but their synergistic effects were not evaluated. Consequently, we believe that these results support the efficacy of such a combination for OA therapy.

Our data revealed more chondrocytes in the articular cartilage of the group treated with SMMSC/secretome /PRP which may be due to the releasing anti apoptotic, antioxidant factors by SMMSC and PRP. Also, we showed that SMMSC/secretome impact on cartilage repair as well that may be related to anti-inflammatory properties of the SMMSC, secretome and PRP content. Along with our findings, it was mentioned that MSCs resulted in articular cartilage improvement in monosodium iodoacetate-induced osteoarthritis rat model through increment in the number of cartilage superficial layer cells. MSCs significantly affect cartilage repair due to their self-renewal capacity, differentiation into various types of host tissue cells, and secretion of critical factors for cartilage regeneration [35]. Additionally, both MSCs and PRP release pro-angiogenic factors, including VEGF and TGF, which improve the subchondral bone vasculature and mobilize the circulation, resulting in decreased cartilage degradation and accelerated KOA repair [36]. Moreover, MSCs can secrete various cytokines that modulate inflammation and exert anti-apoptotic and antioxidants activities. During cell culture, the paracrine factors released by MSCs concentrate in the conditioned media. Secretome has been shown to contain transcriptional regulatory factors that drive bone remodeling and growth factors that facilitate osteoblastic differentiation and bone regeneration [37]. Furthermore, studies have shown that MSC/secretome can significantly reduce the expression of inflammation-related agents in the course of OA [38, 39].

We showed that adding PRP to our indictable remedy led to an increase in the reparative properties of SMMSC and secretome. In parallel with our study, Ragab et al. (2021) presented that the treatment of rat osteoarthritis using PRP results in reduction of destruction cartilage [40]. Growth factors in PRP promote host cell proliferation and migration. Furthermore, Moussa et al. (2017) studied the effect of PRP on osteoarthritic chondrocytes resulting in augmentation of chondrocytes proliferation, collagen, and aggrecan while reduction in MMP-3[41]. Meanwhile, Collagen II expression increment, cartilage surface loss and alleviation in the articular cartilage devastation were reported in those rats osteoarthritis which treated with PRP [42]. Also in accordance with the current results, smooth articular surface and normal joint space in radiography evaluation, articular cartilage hyper cellularity and returning of the articular cartilage to normal thickness based on H&E staining and also moderate matrix staining according to Safranin O stain were observed in rats with induced-OA treating using PRP comprising diverse types of storage molecules for tissue healing and simplifying tissue regeneration [43].

Kim et al. in 2020 conducted a study to evaluate the therapeutic effects of intra-articular synovial derived-MSC injections in a canine OA model. Histopathological analyses including H&E, Safranin-O, Toluidine blue, and Masson’s trichrome showed that cartilage structure and proteoglycan staining were significantly improved in MSCs-treated group in comparison with others [44]. Our data confirms this finding as well.

MMPs are zinc and calcium-dependent endopeptidases that can proteolysis ECM components and play a role in cartilage degradation by degrading cell surface proteins. OA is thought to be caused by a lack of proteoglycans and a deterioration of the collagen meshwork. MMP-3 is a protease that degrades ECM elements such as proteoglycan and collagen, hence obliterating cartilage matrix formation. Consistent with our findings, Kusuma et al. (2020) showed that KOA treatment with Wharton's Jelly-derived MSCs increased collagen type II expression and decreased MMP3 as a matrix degradation mediator [45]. The data from the current study confirmed that administration of the SMMSC led to a decrease in MMP3 expression along with an increase in proteoglycan and collagen II content that may be due to a reduction in MMP3. The data also showed PRP and CM supplementation had synergistic impact on increasing these factors.

We showed that SOX9 increased by SMMSC/ secretome /PRP administration. The Sox9 gene is demonstrated to be expressed simultaneously with the collagen type II gene during chondrogenesis. The Sox9 protein targets the collagen type II gene and inhibits chondrocyte apoptosis, suppresses IL-1β-induced proliferation, reducing aggrecan and collagen type II breakdown, and alleviating OA symptoms. In addition, Sox9 makes normal metabolism possible for cartilage cells in a medium with a slight lack of blood supply and hypoxia. Collagen provides the tissue with tensile strength. Therefore, increase in collagen II in the articular cartilage of the group treated with SMMSC/secretome /PRP may be due to inducing SOX9 expression. One of the major proteoglycans, aggrecan, increases cartilage' water absorption, improves articular cartilage mobility, and alleviates joint discomfort. Zhi et al. (2020) utilized bone marrow-derived MSCs to treat osteoarthritis in rats and observed significant increases in aggrecan, collagen type II, and SOX9 expression [46]. Our data also confirmed this previous finding. Furthermore, Luting et al. (2021) demonstrated that PRP treatment for rabbit knee osteoarthritis resulted in stimulation the expression of Sox9. In addition, Sox9 makes normal metabolism possible for cartilage cells in light of a lack of blood supply and hypoxic medium [47].

The data of the present study indicates that both secretome and PRP reinforce the beneficial impacts of SMMSC. PRP treatment for osteoarthritis in rabbit knees also increased Sox9 expression, as shown by Luting et al. [47]. In 2019, Chen et al. reported that treatment of osteoarthritis in rat using secretome of human bone marrow‐derived MSCs resulted in smooth and regular articular surface, and also preservation of GAGs and collagen II [6]. In addition, Khatab et al. (2018) stated pain reduction and protective efficacy on the evolvement of cartilage destruction after human bone marrow-derived MSCs injection in a mouse collagenase-induced OA model [48]. Clinically, increment of collagen II expression and reducing MMP-3 activity were reported in patients with OA treated using conditioned media from human adipose-tissue-derived MSCs [49]. Furthermore, Tong et al. (2020) demonstrated that umbilical cord-derived MSCs treatment in rat-induced OA deferred chondrocytes apoptosis, promoted cartilage superficial layer cells and preserved cartilage structure [50].

Based on the current experimental research, several limitations which need to be investigated in the future are as follows: First, evaluation using molecular tests to show the cellular functions and changes. Second, more in-depth investigations are essential in terms of angiogenesis, prevention of wear of the cartilage and inhibition of osteophyte formation. Last, it’s necessary to perform this study in larger animals before clinical usage.

## Conclusions

The current study revealed the important role of combination of the synovial membrane-derived MSCs with PRP and secretome in the KOA treatment. In addition, betterment in KOA in terms of joint space, articular cartilage structure, sub-chondral bone and ECM constituents were observed through inhibition the expression of MMP-3, and promotion the collagen II and Sox9 expression. In conclusion, injecting the combination of the synovial membrane-derived MSCs with PRP and secretome could be considered as a promising therapy for KOA patients.

## Data Availability

All data generated or analyzed during this experimental study are included in this published article.

## Abbreviations

SMMSCs: Synovial membrane-derived MSCs
PRP: Platelet-rich plasma
OA: Osteoarthritis
KOA: Knee osteoarthritis
TGF-β: Transforming growth factor
PDGF: Platelet-derived growth factor
PAP: Plasma average platelet
DMEM: Dulbecco’s modified Eagle’s medium
MMP: Matrix metalloproteinase
GAGs: Glycosaminoglycans
SOX: SRY-related high-mobility group (HMG) box
ECM: Extracellular matrix
IL-1β: Interleukin 1 beta
IGF: Insulin-like growth factor
TNF: Tumor necrosis factor
VEGF: Vascular endothelial growth factor
H&E: Hematoxylin and eosin
